# *Rickettsia aeschlimannii* in *Hyalomma marginatum* Ticks, Germany

**DOI:** 10.3201/eid1702.100308

**Published:** 2011-02

**Authors:** Leonid Rumer, Elmara Graser, Timo Hillebrand, Thomas Talaska, Hans Dautel, Oleg Mediannikov, Panchali Roy-Chowdhury, Olga Sheshukova, Oliver Donoso Mantke, Matthias Niedrig

**Affiliations:** Author affiliations: Robert Koch Institut, Berlin, Germany (L. Rumer, P. Roy-Chowdhury, O. Sheshukova, O. Donoso Mantke, M. Niedrig);; AJInnuscreen GmbH, Berlin (E. Graser, T. Hillebrand);; Practice for Microbiology and Epidemiology of Infectious Diseases, Lindow, Germany (T. Talaska);; IS Insect Services GmbH, Berlin (H. Dautel);; Université de la Méditerranée, Marseille, France (O. Mediannikov)

**Keywords:** Vector-borne infections, bacteria, Rickettsia aeschlimannii, spotted fever group, ticks, Hyalomma marginatum, Germany, rickettsia, letter

**To the Editor:** *Rickettsia* spp. of the spotted fever group cause worldwide emerging human infections known as tick-borne rickettsioses (*1*). Data on the occurrence and prevalence of *Rickettsia* in Germany are still limited (*2*). Six *Rickettsia* species have been reported to date (*2*). *R. helvetica, R. felis, R. massiliae*, and *R. monacensis* were detected with a relatively low prevalence in *Ixodes ricinus* ticks collected in southern Germany (*2*); *R. raoultii* was identified with high prevalence in the rapidly expanding area where *D. reticulatus* ticks are found (*2*). *R. raoultii* was recently recognized as an agent of tick-borne lymphadenopathy/*Dermacentor*-borne necrosis and erythema lymphadenopathy (*3*). Low prevalence of another tick-borne lymphadenopathy agent, *R. slovaca,* in *Dermacentor marginatus* ticks collected in southern Germany was recently reported (*4*).

We report the detection in Germany of the pathogenic SFG species *R. aeshlimannii* (*1*), which is phylogenetically close to *R. raoultii* and causes an infection with clinical signs similar to those of Mediterranean spotted fever (*1*). To determine the prevalence of *R. raoultii* in the ticks in Berlin/Brandenburg and neighboring regions, we collected 294 ticks; 288 had been collected either from vegetation or domestic animals and morphologically identified as adult *D. reticulatus* ticks. The remaining 6 ticks were delivered by an ornithologist who had removed them from a bird (belonging to the *Acrocephalus scirpaceus* spp.) that he had captured in the reeds near Pakendorf and Zerbst, Saxony-Anhalt, in May 2007. These 6 ticks were reported as *D. reticulatus*–like adults but were damaged in the collection process, making an exact morphologic evaluation impossible.

DNA was isolated from the complete tick body by homogenization in the SpeedMill (Analytik Jena Biosolutions, Jena, Germany) followed by purification by RapideStripe tick DNA/RNA Extraction Kit (Analytik Jena Biosolutions). Multispacer typing (*5*) was used for molecular detection and determination of *Rickettsia* spp. (Figure). DNA sequencing and analysis were performed as described (Figure).

**Figure:**
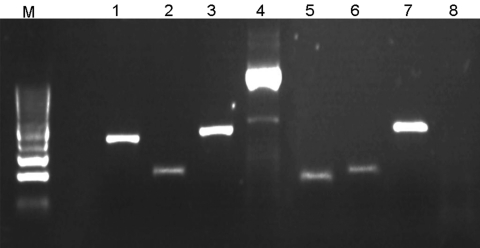
Illustration of multispacer typing. Amplicons 1–4 result from PCRs on DNA obtained from 1 *Rickettsia raoultii*–infected *Dermacentor reticulatus* tick isolate; lanes 5–8 result from PCRs on 1 damaged isolate. PCRs amplifying dksA-xerC (lanes 1 and 5), mppA-purC (lanes 2 and 6), and rpmE-tRNA (lanes 3 and 7) intergenic spacers were performed as described (*5*). PCR amplifying the entire internal transcribed factor 2 (ITS2) locus of *D. reticulatus* tick (lanes 4 and 8) was involved in each PCR run as a positive control and for validation of *D. reticulatus* tick identity (the primers will be described elsewhere).The negative result of ITS2 PCR with the damaged isolates (lane 8) indicated that they are not *D. reticulatus* ticks. Lane M, DNA size marker (100-bp ladder). PCR products were directly sequenced in both directions with respective primers by an ABI PRISM DNA Sequencer (Applied Biosystems, Foster City, CA, USA). DNA Star package (DNA Star, Madison, WI, USA) and the tools offered by the National Center for Biotechnology Information (www.ncbi.nlm.nih.gov) were used for DNA search and analysis.

In 51.3% of the intact tick isolates, *R. raoultii* was detected. In each of the 3 damaged isolates, the multispacer type pattern was detected, which was 100% identical to that of *R. aeschlimannii* (*5*) (Figure). Moreover, PCR, which we routinely use as a positive control for molecular identification of *D. reticulatus*, yielded no product in the damaged isolates (Figure).

To determine the species of the damaged ticks, we used 3 tick-specific PCRs. One amplified a 16S rRNA fragment used for phylogenetic studies of ticks (*6*). Use of the other 2 PCRs was based on the consideration that *R. aeschlimannii* is usually found in ticks of the genus *Hyalomma,* primarily in *H. marginatum* (*1*). Therefore, 1 of the PCRs amplified a fragment of the *Hyalomma* tick mitochondrial cytochrome oxidase I gene and the other a fragment of the internal transcribed spacer 2 (*7*).

The ITS2 fragment displayed the highest (99%) similarity with the respective fragment of *H. marginatum*, *H. dromedarii*, *H. truncatum,* and *H. lusitanicum.* Cytochrome oxidase subunit I fragment was 99% identical to *H. marginatum*, *H. dromedarii*, and *H. truncatum*. The 16S RNA fragment was 98% identical to *H. marginatum*; its identity to the second closest sequence belonging to *H. lusitanicum* was 96%.

Earlier, *R. aeschlimannii* had been detected in sub-Saharan and North Africa, southern Europe, and southwestern Russia (*8*). Therefore, the area of Zerbst, the middle of Germany, marks the northernmost point of *R. aeschlimannii* detection.

*Hyalomma* spp. ticks are distributed in Africa, the Mediterranean climatic zone of southern Europe, and in Asia. The only documented *Hyalomma* spp. tick in Germany was found on a human in the southern part of the country (Lake Constance area) in May 2006, but the possibility of tick transportation from Spain was not ruled out (*9*).

*Acrocephalus scirpaceus* birds are migratory birds and live in central Europe from April to October and winter in sub-Saharan Africa in the region inhabited by *Hyalomma* spp. ticks. Therefore, it is reasonable to suggest that the *Hyalomma* spp. ticks that we examined had been transported by the birds from Africa. The fact that a randomly caught bird was infested with *R. aeshlimannii*­–infected ticks is suggestive of the intensive stream of new pathogens transported through Europe by migrating birds. The first possible implication of a bird as a vector of infected ticks was proposed for *Hyalomma* spp. ticks infected by *R. sibirica mongolitimonae* (*10*). Until now, the role of migrating birds in distribution of tick-borne pathogens has been poorly understood (*9*). The changing climate and environment in central Europe may facilitate the establishment of pathogen-carrying tick species transported by birds. These new pathogens can be directly transmitted from infected birds to the species of the local fauna.
